# Supplementary material for: Patterns of Intron Gain and Loss in Fungi
Source: PLoS Biol. 2004 Nov 30;2(12):e422. doi: 10.1371/journal.pbio.0020422 (PMC532390; doi:10.1371/journal.pbio.0020422)
Supplement: Table S1 — Also available at http://genes.mit.edu/NielsenEtAl/. (4.3 MB ZIP). [file pbio.0020422.st001.zip › NielsenEtAl/html/392.html]

AN5808.1.NCU07705.1.MG01734.1.FG05068.1


```
 CLUSTAL W (1.82) Multiple Sequence Alignments - Introns Inserted


Sequence 1: NCU07705.1	677 aa
Sequence 2: MG01734.1	698 aa
Sequence 3: FG05068.1	696 aa
Sequence 4: AN5808.1	632 aa
Alignment Length: 733 aa
Number Identitical Residues: 314 aa
Alignment Score (without introns) 16275


MG01734.1 	MSPSSTGESPPTILNGHSAPAVSNG---RGFPLPSRDTFNIDIPTSQLN-TSNPPSALKS
NCU07705.1	MSPSSNSDSPASVVGSMTMAAQAAPNVVRQYPLPNRD-FTIEIPSPQVAPTTNGVNTVKS
FG05068.1 	MSPSSTGESAHSISINS----------ARPFTVPARDSFALDLPMSSLA-TMSADASLKS
AN5808.1  	MSSS-----------------------------PERD-----------------------
          	**.*                             * **                       

MG01734.1 	PATVKSQRTPSFSRDSILGSALKAGASQRADFS-GDGRLGDPNGLQKVSSDEGSNPLKRR
NCU07705.1	PSTLKSSRAPNFSREGILGSAQKARNLSQSSEN-RPDHVNVSNGFHKAPSEESTNPLKRR
FG05068.1 	PVSYKNQRTPSFS----MVTSQKARHLSQSSDNNRPD--NMSNGGQKVSSDEGSNPLKRR
AN5808.1  	----------------------REGILDSTSAS----------------LNELIN--KKR
          	                      :    . :. .                 :*  *  *:*

MG01734.1 	NTDAGVDYPRRRATIA0CEVCRSRKSRCDGTKPKCKLCLELGADCVYREPGIKLDAGDKL
NCU07705.1	NTDAGVDYPRRRATIA0CEVCRSRKSRCDGTKPKCKLCTELGAECIYREPGIKLDAGDKL
FG05068.1 	NTETGVDYPRRRATIA0CEVCRSRKSRCDGTKPKCKLCTELGAECIYREPGIKLDAGDKL
AN5808.1  	SNSEATEYPRRRATIA0CQICRLRKTRCNGARPKCQLCTDLDAECVYREPGIKLDAGDKL
          	... ..:********* *::** **:**:*::***:** :*.*:*:**************

MG01734.1 	ILERLNRIESMLQMTLPPN----GIGLGG-SPNLSNGTGHLSADGILTGGP----FLSVM
NCU07705.1	ILERLNRIESLLQLNMGLTGHTHGLGLSHNSPGMSNGTG-LSGDNLMMQNPDAAGFVSVI
FG05068.1 	ILERLNRIESLLQMSMVNTTAN-GLGLTENSPGMSNGTG-LDGDNLILSAN--AATFSSI
AN5808.1  	IIERLARIEGLLQTSLAAQNSQ--LTLSSTSPATSNGTNVGSDDTVAKASTHLAVTGRTT
          	*:*** ***.:** .:        : *  .**  ****.  . * :       :      

MG01734.1 	PNAALSQW-ATAPN--ISTMPKVHTNAALHLLQWPLIRELVSRPYDPQILLQLEMAREPL
NCU07705.1	PSGGLGTWSATATNS-LSTMPKVHTNAALHLLQWPMIRDLVSRPYDPQILLQLEMAREPL
FG05068.1 	PNGGFGTWSAQPVGTNISTMPKVHTNAAMHLLQWPLIRDLISRQCDPQVLLQLEMAREPL
AN5808.1  	SIVGLASWANPPTS--ISTMPRVHTTPALNLLKWPLIRDLVARPYDSQTLLQLEMAREPL
          	.  .:. *:  . .  :****:***..*::**:**:**:*::*  *.* ***********

MG01734.1 	HSLTKTPCVDLSNTAAYIEAYFERVNVWYACVNPYTWRSHYRIALSNGFREGPESCIVLL
NCU07705.1	HSLTKTPCVDLSNTSAYIEAYFDRVNVWYACVNPYTWRSHYRIALSNGFREGPESCIVLL
FG05068.1 	HTLTKTPCVDLSNTQAYIEAYFERVNIWYACVNPYTWRSQYRTALSNGFREGPESCIVLL
AN5808.1  	K-MTPPMAVDLSCAPLYIRTFFEKVNVWYACVNPYTWSKYYDAAVSLSFREGPESCVVLL
          	: :* . .**** :  **.::*::**:********** . *  *:* .********:***

MG01734.1 	VLALGQASLKGSISRIVPHEDPPGLQYFTAAWALLPGMMTSNSVLAAQSHLLAAAYLFYL
NCU07705.1	VLALGQASLRGSISRIVPHEDPPGLQYFTAAWALLPGMMTSNSVLAAQCHLLAAAYLFYL
FG05068.1 	VLALGQASLHGSISRILPHEDPPGLQYFTAAWSLLPGLMTANSVVAAQCHLLAAAYHFYL
AN5808.1  	VLALGCAAQCGSIALISTDRDPPGVPYFIAAWNLLPSVMMRNSVVAAQCIILAAAYLFYL
          	***** *:  ***: * ...****: ** *** ***.:*  ***:***. :***** ***

MG01734.1 	VRPLEAWNLLCTTSTKLQLLLMSPSRVPSDQRELTERIYWNSLLFESDLLAELDLPHSGV
NCU07705.1	VRPLEAWNLLCTTSTKLQLLLMTPNRVPVDQRELSERIYWNALLFESDLLAELDLPHSGV
FG05068.1 	VRPMEAWNLLCTTSTKLQLLLMAPSRIPSHQRELVERIYWNALLFESDLLAELDLPHSGV
AN5808.1  	VRPLEAWTLLSNASMKLQLLFGNPSRVPVQWRELSVRVYWNALLYESDLLAELDLPHSGI
          	***:***.**..:* *****:  *.*:* . ***  *:***:**:**************:

MG01734.1 	VQFEENVGLPGGF---EGDENE-----------AVGRDDLWYFLAEIALRRLLNRVSNLI
NCU07705.1	VAFEENVGLPCGF---EGDEQE-----------AVGRDDLWYFLAEIALRRLLNRVSQLI
FG05068.1 	VQFEEHVGLPGGF---EGDESE-----------QVGRDELWYFLAEIALRRLLNRVSHLI
AN5808.1  	VNFEELVDLPGGFDEIEDDEDEDDGDTGNKLVSPIGCDELWYFLAEIALRRLLNRVSHML
          	* *** *.** **.. *.**.*....:...  : :* *:******************:::

MG01734.1 	YSKDSMASTTSLEPVVAELDFQLTQWYESLPLPLQFPFTRTMLPDPVQTVLRLRFFACRT
NCU07705.1	YSKDSLASTTSLEPIVTELDFQLTQWYESLPLPLQFPFTRTMLPDPVQTVLRLRFFACRT
FG05068.1 	YSKDSIANTQSLDPVVAELDYQLSQWYESLPLPLQFPFTRNALQDPVQTVLRLRFFACRT
AN5808.1  	YQKDNSLTLASLAPIVSELDFQLSQWYEGLPQPVRFPLSHAPISHPVQTVLRLRYFACRT
          	*.**.  .  ** *:*:***:**:****.** *::**:::  : .*********:*****

MG01734.1 	IIYRPYILAVLDNEQAVLDPAVRDSCHKCLEASIRQLEHIAAH2HAGHIPYLWQGALSIV
NCU07705.1	IIYRPYILAVLDNEQAILDPSVRDACTKCLEASIRQLEHISAH2----------------
FG05068.1 	IIYRPYILAVLDNEQQVLDPAVRENCHKCLEASLRQLEHIHEH2HAGHMPYIWQGALSMV
AN5808.1  	IIYRPYILAVFENEQASGDSWVRECCRRCLEATLYQLESVTTH~REGHLPYLWQGALSMV
          	**********::***   *. **: * :****:: *** :  *   .  .   ..: :  

MG01734.1 	SQTLLVMGATMSPSLSSILSTVVPHRETLDQILSEVIIEIERYAVLAPSLSLAADILKEA
NCU07705.1	-----------------ILVSLVSHRDVIDQIINDVVMEIERYSVLSPSLSLSAEIIKEA
FG05068.1 	SQTLLIMGATMSPSLLNILLTLVPSREALDQIINDVVMEVERYATLAPSLSLASEMLKEA
AN5808.1  	SQSLLIMGATMSP----ALSSLLPPTLRVDEIISSVIVEVDRYAHLAPSLKLSSEILRDA
          	:.:    .:: :.     * :::.    :*:*:..*::*::**: *:***.*:::::::*

MG01734.1 	E~VRRRAFLMSG----
NCU07705.1	E~VRRRTFLSG-----
FG05068.1 	E0YCFSIISPRVLD--
AN5808.1  	E~RRRQICLRSSGSRV
          	*            .
```
